# Reproducibility of Heart Rate Variability Is Parameter and Sleep Stage Dependent

**DOI:** 10.3389/fphys.2017.01100

**Published:** 2018-01-10

**Authors:** David Herzig, Prisca Eser, Ximena Omlin, Robert Riener, Matthias Wilhelm, Peter Achermann

**Affiliations:** ^1^Preventive Cardiology and Sports Medicine, University Clinic for Cardiology, Bern University Hospital (Inselspital), University of Bern, Bern, Switzerland; ^2^Sensory-Motor Systems Lab, Institute of Robotics and Intelligent Systems, ETH Zurich, Zurich, Switzerland; ^3^Medical Faculty, University of Zurich, Zurich, Switzerland; ^4^Institute of Pharmacology and Toxicology, Zurich Center for Interdisciplinary Sleep Research and Zurich Center for Integrative Human Physiology, University of Zurich, Zurich, Switzerland

**Keywords:** sleep stages, heart rate, heart rate variability, reproducibility, autonomic nervous system, slow wave sleep

## Abstract

**Objective:** Measurements of heart rate variability (HRV) during sleep have become increasingly popular as sleep could provide an optimal state for HRV assessments. While sleep stages have been reported to affect HRV, the effect of sleep stages on the variance of HRV parameters were hardly investigated. We aimed to assess the variance of HRV parameters during the different sleep stages. Further, we tested the accuracy of an algorithm using HRV to identify a 5-min segment within an episode of slow wave sleep (SWS, deep sleep).

**Methods:** Polysomnographic (PSG) sleep recordings of 3 nights of 15 healthy young males were analyzed. Sleep was scored according to conventional criteria. HRV parameters of consecutive 5-min segments were analyzed within the different sleep stages. The total variance of HRV parameters was partitioned into between-subjects variance, between-nights variance, and between-segments variance and compared between the different sleep stages. Intra-class correlation coefficients of all HRV parameters were calculated for all sleep stages. To identify an SWS segment based on HRV, Pearson correlation coefficients of consecutive R-R intervals (rRR) of moving 5-min windows (20-s steps). The linear trend was removed from the rRR time series and the first segment with rRR values 0.1 units below the mean rRR for at least 10 min was identified. A 5-min segment was placed in the middle of such an identified segment and the corresponding sleep stage was used to assess the accuracy of the algorithm.

**Results:** Good reproducibility within and across nights was found for heart rate in all sleep stages and for high frequency (HF) power in SWS. Reproducibility of low frequency (LF) power and of LF/HF was poor in all sleep stages. Of all the 5-min segments selected based on HRV data, 87% were accurately located within SWS.

**Conclusions:** SWS, a stable state that, in contrast to waking, is unaffected by internal and external factors, is a reproducible state that allows reliable determination of heart rate, and HF power, and can satisfactorily be detected based on R-R intervals, without the need of full PSG. Sleep may not be an optimal condition to assess LF power and LF/HF power ratio.

## Introduction

There is growing interest for assessments of the activity of the autonomic nervous system (ANS) both in research and in applied settings. Few methods exist to measure activity of certain branches of the ANS, such as microneurography to assess muscle sympathetic nerve activity or determination of noradrenalin in the blood to indicate spill-over of the sympathetic nervous system (Grassi and Esler, 1999). HRV is a widely used indirect marker of the cardiac autonomic nervous system activity (CANA) and finds applications in different fields such as psychology (Laborde et al., 2017), sport science (Aubert et al., 2003; Buchheit et al., 2004; Bellenger et al., 2016; Plews et al., 2016) and medicine, namely prenatal diagnostics (Fairchild, 2013), sleep studies (Tobaldini et al., 2013; Dodds et al., 2017), and mortality risk assessment in diseased patients (Huikuri, 1995; Malik et al., 2016; Zhou et al., 2016). In contrast to the other methods, HRV offers a non-invasive and simple method to approximate the CANA.

A fundamental constraint of HRV measurements is the rather poor reproducibility that can be improved when measurements are highly standardized. Standardized measurements are usually performed during 2–5 min in supine position after resting for at least 10 min at the same time of the day (Camm, 1996). However, this time requirement may be problematic in situations where regular (daily) measurements are needed, such as monitoring fatigue and training adaptations in athletes (Plews et al., 2014). Further, resting HRV measurements may also be problematic in young children who cannot stay motionless and relaxed on demand.

Recently, studies have analyzed HRV during sleep to investigate effects of acute stress (Hall et al., 2004; Hynynen et al., 2011), chronic stress (Hynynen et al., 2006), physical activity (Herzig et al., 2017), or diseases (Roumelioti et al., 2010; Amra et al., 2017) on the CANA. Indeed, sleep could provide a highly standardized condition to time-efficiently measure HRV. However, sleep architecture has been shown to affect the CANA in various ways (Somers et al., 1993; Silvani and Dampney, 2013), and also HRV parameters have been shown to vary between different sleep stages and as a function of circadian phase (Trinder et al., 2001; Busek et al., 2005; Vandewalle et al., 2007; Boudreau et al., 2013). Compared to wakefulness, sleep is characterized by a generalized cardiovascular deactivation and a resetting of baroreflex sensitivity (Silvani and Dampney, 2013). Rapid-eye movement (REM) sleep exhibits relatively high muscle sympathetic nerve activity, high lumbar sympathetic nerve activity, and low muscle tone (Silvani and Dampney, 2013). During REM sleep, levels of sympathetic nerve activity above wakefulness values have been recorded when bursts in sympathetic nerve activity induce blood pressure surges and sudden increases in heart rate (Somers et al., 1993). During non-rapid-eye movement (NREM) sleep, low sympathetic activity (Somers et al., 1993; Silvani and Dampney, 2013), reduced cardiac output and lower blood pressure (Somers et al., 1993) have been observed. In sleep stage 2, the occurrence of arousal stimuli accompanied by bursts of sympathetic nerve activity induce a transient increase in blood pressure and heart rate (Trinder et al., 2003). The amplitude and frequency of sympathetic-bursts and blood pressure surges are lower in slow-wave sleep (SWS, N3) than in stage 2 (Somers et al., 1993). SWS is a standardized state with reduced blood pressure variability (Silvani, 2008), constant autonomic activity and a regular breathing frequency, undisturbed by emotional stimuli (Murali et al., 2003). Hence, the absence of sympathetic activity bursts and stationarity of heart rate during SWS offers a highly standardized condition for HRV assessment (Brandenberger et al., 2005). While measurements during SWS are appealing, their validity and usefulness have only sparsely been investigated. In particular, the reliability of HRV measurements has, to our knowledge, never been investigated during specific sleep stages.

Determination of SWS usually requires PSG recordings. However, previous studies have shown close correspondence of HRV parameters and cortical activity (Charloux et al., 1998; Otzenberger et al., 1998; Brandenberger et al., 2001; Dumont et al., 2004). Further, specific HRV characteristics in SWS have been observed, with stationary heart rate and uncorrelated consecutive R-R intervals (Herzig et al., 2016). Recent studies have attempted to classify sleep stages using ECG parameters only (Ebrahimi et al., 2013; Long et al., 2014, 2017; Fonseca et al., 2015; Yoon H. et al., 2017; Yoon H. N. et al., 2017). Moreover, other studies have used a simple method to identify a segment within SWS and analyze HRV (Al Haddad et al., 2009; Herzig et al., 2016, 2017). However, the reliability of these latter methods, relying solely on HRV parameters to select a segment located in a SWS phase, without necessitating PSG recordings, have never been investigated. This method would allow simple HRV measurements in a standardized state that is least influenced by environmental factors.

The aims of this study were to assess the reproducibility of HRV parameters during the different sleep stages by quantifying the variances of HRV parameters between and within subjects, as well as between and within nights of individuals. Furthermore, using the information of PSG sleep staging, we verified the reliability of an algorithm based solely on HRV data to identify a single 5-min SWS segment within each night.

## Methods

### Subjects

Healthy young males were recruited for a study investigating the effect of vestibular stimulation by a rocking bed on sleep and the sleep EEG (unpublished data). The study was approved by the Institutional Review Board of the Swiss Federal Institute of Technology in Zurich. Written informed consent was provided by all subjects and the study was conducted in accordance with the Declaration of Helsinki.

### Study design

In the original study, the effect of two different bed rocking protocols on sleep onset was investigated. After a familiarization night, three nights were recorded in all subjects, two with rocking and one with a control condition (randomized cross-over design). The bed rocking protocols were: no rocking, rocking from lights off until sleep onset and rocking from before sleep onset until the end of the first 2 h of sleep. Due to the absence of a significant treatment effect on the mean and variance of any of the analyzed parameters (sleep onset, HRV parameters, heart rate), the data were pooled for the present analyses.

All participants underwent a screening night (polysomnography, PSG) to rule out sleep disorders prior to the study. They were normal sleepers (habitual sleep duration ~8 h) with moderate alcohol and caffeine consumption. Participants had to adhere to regular bed times for 7 days prior to the first experimental night and throughout the entire experimental phase (3 weeks in total). Furthermore, they had to abstain from caffeine and alcohol consumption during the 3 days prior to each experimental night. Compliance with the bedtimes was assessed by actigraphy and sleep logs. Details on the methodology will be published elsewhere.

### Measurements

#### PSG recordings/sleep staging

PSG data (EEG, EOG, EMG, ECG, and respiration) were continuously recorded throughout the entire 8-h sleep period with a polygraphic amplifier Artisan (Micromed, Mogliano, Veneto, Italy). The signals were sampled at 256 Hz and recorded with the software Rembrandt DataLab (Version 8.0; Embla Systems, Broomfield, CO, USA). The analog signals were filtered with a high pass filter (EEG: −3 dB at 0.16 Hz; EMG: 10 Hz; ECG: 1 Hz) and an anti-aliasing low-pass filter (−3 dB at 67.2 Hz). For further analysis, the EEG signals were re-referenced to the mastoids (A1, A2). The sleep stages were scored visually on a 20-s epoch basis as suggested by Rechtschaffen and Kales (Rechtschaffen and Kales, 1968) according to standard criteria (Iber et al., 2007). For artifact removal, artifacts were identified visually and with a semi-automatic artifact detection (see Lustenberger et al., 2012 for details).

### HRV analysis

**Figure 1:**
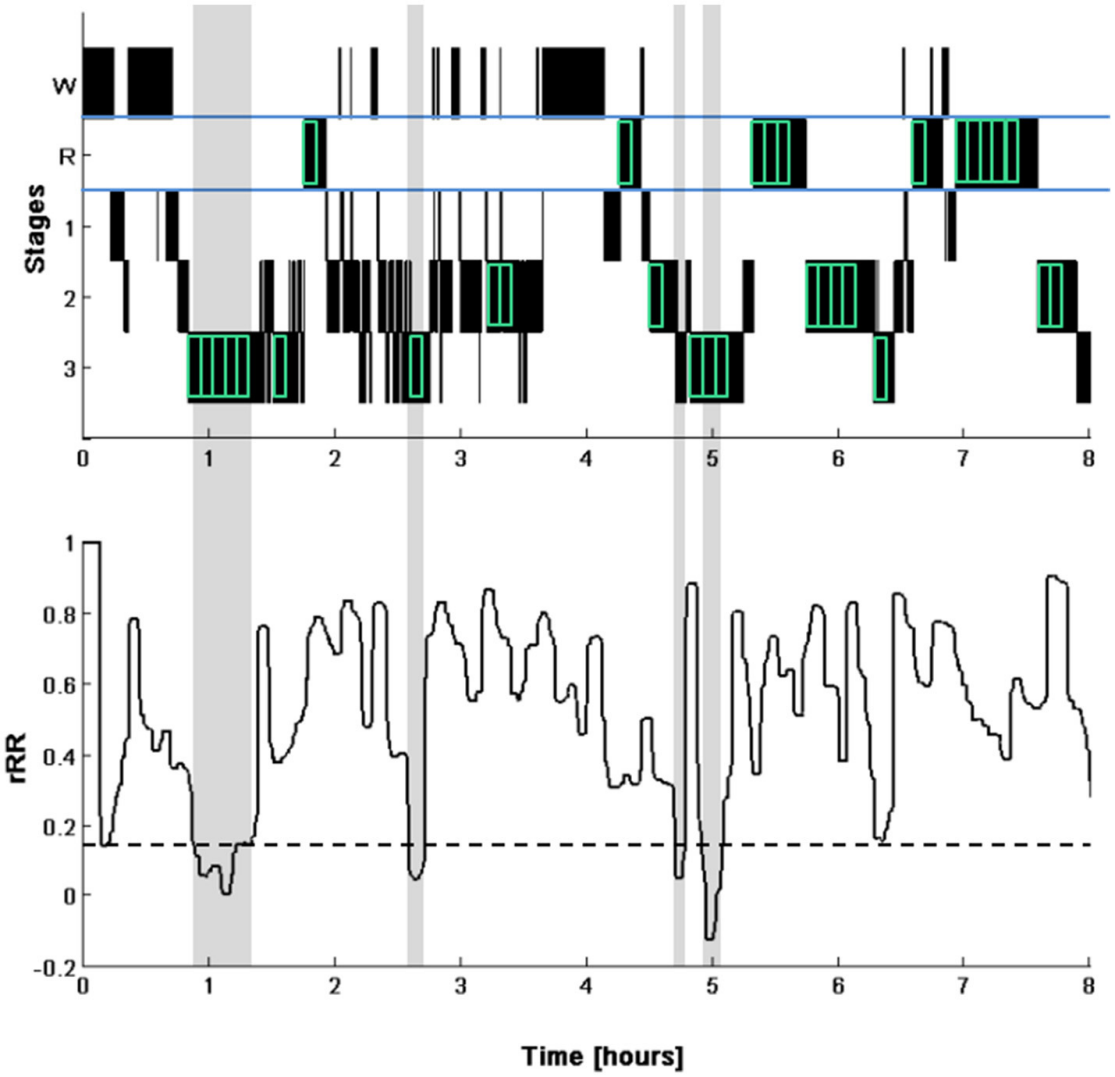
Illustration of the selection of the segments for HRV analysis **(top)**. Hypnogram (W, waking; R, REM sleep; 1 to 3, NREM sleep stages N1 to N3). The segments selected for the analysis are indicated by the green boxes (indicated for visual purpose only and are not to scale). Note that the last 5-min segments of a continuous phase of a particular sleep stage were discarded. Time course of the correlation of consecutive R-R intervals (rRR, **bottom**) calculated with a 5-min windows moved in steps of 20 s. The dotted line represents the cut-off for determining periods of SWS (0.1 unit below the mean of the first 4-h). The identified SWS phases are highlighted in gray. Note that only one 5-min segment in the middle of the first detected SWS phase was used for our analyses.

R-peaks were automatically detected from the ECG trace using the ≪nqrsdetect≫ function from the Biosig Matlab (2014a, The Mathworks, Natick, MA) Toolbox (Vidaurre et al., 2011) and inter-beat durations (R-R intervals) were calculated. R-R intervals were analyzed using a Matlab procedure specifically developed for this study. After a stage change, only consecutive 5-min segments consisting of a particular stage were used for analysis (Figure 1). Due to the often reported time delay of varying length between CANA and cortical activity (Jurysta et al., 2003; Long et al., 2015) (with CANA preceding cortical activity), the last 5-min segment of each sleep phase was discarded. Thus, only sleep phases longer than 10 min were included in the analysis. In each 5-min segment, frequency domain and time domain parameters were calculated. Mean heart rate [beats per minute, bpm] was derived from the mean of the R-R intervals using the following formula: heart rate [bpm] = 60,000/(mean R-R intervals [ms]). For spectral analysis, R-R intervals were interpolated (cubic spline interpolation) and resampled at 4 Hz. We applied an advanced smoothness prior approach for detrending of the R-R intervals with a smoothing parameter of λ = 500, which corresponds to a high pass filter with a cut-off frequency of 0.035 Hz (Camm, 1996). We used an artifact correction algorithm that eliminates R-R intervals in case of deviations of 30% or more to adjacent R-R intervals and replaced them using a cubic-spline interpolation. Power spectral density was then calculated using the Welch method (1967) with a Hamming window length of 128 points and an overlap of 50%. Frequency domain parameters were total power (TP, ms^2^, 0–0.4 Hz), low-frequency power (LF, ms^2^, 0.04–0.15 Hz), high-frequency power (HF, ms^2^ 0.15–0.4 Hz), and the LF/HF ratio. The following time domain parameters were calculated: the square root of the mean square differences of adjacent R-R intervals (RMSSD, ms) and the standard deviation of all R-R intervals (SDNN, ms). Normalized frequency parameters are not reported in order to avoid redundancy with LF/HF ratio (Massin et al., 1999; Burr, 2007).

### SWS segment identification using HRV (segment_low rRR_)

By means of a custom built Matlab script, Pearson's correlation coefficient in the Poincaré plot of the consecutive R-R intervals (rRR, Brennan et al., 2001) were calculated of 5-min windows moved in steps of 20 s over the whole night. The linear trend of rRR time series in the first 4 h was removed and we identified the first period where rRR values were 0.1 units below the mean rRR (which was 0 due to the detrending) of the first 4 h for at least 10 min, the same threshold has been used in a previous study (Herzig et al., 2016). A 5-min segment (*Segment*_*low rRR*_) was then put in the middle of the identified period (mid-point of 5-min segment allocated to the midpoint of the identified period) of which frequency and time domain analysis of the R-R intervals were performed. Due to the detrending procedure all segments were placed in the first 4 h of the night.

### Verification of the identified SWS segment (segment_low rRR_)

For each night, the *Segment*_*low rRR*_ (identified by means of the HRV signal) was classified as accurately detected if the complete 5-min segment was located in a phase of SWS (as classified according to the PSG recordings). Further, segments consisting of at least 50% SWS were counted and reported separately.

### Statistical analysis

In order to quantify the variances of heart rate and HRV parameters of the different sleep stages, segments were grouped according to sleep stage as identified by PSG. The study design with three nights within each subject and several segments of different sleep stages within each night allowed the following variance partitioning. Total variance was partitioned into: Between-subjects variance (Var_between Sub_), between-nights variance (Var_between Nights_), and between-segments variance (Var_between Segments_) with total variance being equal to the sum of theses variances (Total Variance = Var_Between Sub_ + Var_Between Nights_ + Var_Between Segments_). Further, the variance components were expressed as proportions of total variance as well as of the within-subjects variance (Within-Subjects Variance = Var_Between Nights_ + Var_Between Segments_). For this purpose, nested random effect models were applied using the R software package lme4 (Bates et al., 2015) to quantify the variances of interest. Heart rate, HF power, LF power, LF/HF ratio, RMSSD, and SDNN were entered as dependent variables. Standard deviations ($\sqrt{Var},\;SD$) were calculated and are reported as a measure of absolute variability.

The between-subjects/within-subjects intra-class correlation coefficient (ICC) was estimated as the proportion of the between-subjects variance to the total variance (ICC = Var_Between Sub_/Total Variance) which is equivalent to using a two-way mixed model analysis of variance and the ICC(3,1) formula as defined by Shrout and Fleiss (Shrout and Fleiss, 1979). For the within-subject reliability, the between-nights/within-nights ICC was calculated (ICC = Var_Between Nights_/(Var_Between Nights_ + Variance_Between Segments_)). ICCs were determined for heart rate and all HRV parameters in all sleep stages. Between-nights/within-nights ICC, providing information on whether different nights of the same subject can be distinguished, corresponds to the proportion of the between-nights variance of the within-subject variance. Interpretation of ICCs was based on the classification by Cicchetti (1994).

Statistical analyses were performed using the software R (Version 3.3.1, R Core Team, 2016). Models were tested with regard to satisfaction of underlying statistical assumptions such as normal distribution of residuals and homoscedasticity. Non-normally distributed variables were log transformed and model assumptions were tested again.

## Results

### Subjects

Eighteen healthy male participants with mean age 23.7 ± 2.5 (*SD*) years were included in the study. Data of three subjects had to be excluded. Reasons were insufficient quality of the ECG recording of two subjects and availability of only two nights in another subject. Thus, 15 subjects with 3 recordings each were included in the analysis. Median values of HRV parameters of separate sleep stages are shown in Table 1. While heart rate was highest during REM sleep, comparable heart rates were found in stage 2 and SWS. Similarly, HF power in stage 2 and SWS was lower than during REM sleep, however, differences were small. The lowest values of LF power, RMSSD, and SDNN were observed in SWS, with low LF power also resulting in a low LF/HF ratio.

**Table 1:** Median and interquartile range of HRV parameters in the different sleep stages.

	**Stage 2** **Median (IQR)**	**SWS** **Median (IQR)**	**REM** **Median (IQR)**	**Segment_low rRR_** **Median (IQR)**
Heart rate (bpm)	51.6 (47.9, 58.3)	51.5 (47.9, 55.3)	53.6 (49.7, 58.3)	51.3 (46.7, 54.2)
HF power (ms^2^)	1095 (660, 1841)	1167 (595, 2438)	1322 (689, 2326)	1203 (681, 2509)
LF power (ms^2^)	1303 (683, 2578)	651 (385, 1199)	2541 (1585, 4001)	613 (350, 1133)
LF/HF ratio	1.11 (0.68, 2.02)	0.51 (0.31, 0.90)	2.02 (1.30, 3.22)	0.45 (0.27, 0.78)
RMSSD (ms)	70.7 (54.1, 91.1)	67.3 (49.2, 97.6)	79.7 (58.7, 109.3)	71.3 (51.8, 101.3)
SDNN (ms)	68.5 (50.9, 94.5)	53.8 (40.7, 71.6)	105.5 (82.6, 134.7)	68.4 (51.3, 97.7)

*IQR, Interquartile range; SWS, slow-wave sleep; REM, rapid eye movement sleep; Segment_low rRR_, Segment automatically detected by HRV; HF, High frequency; LF, Low frequency; RMSSD, square root of the mean square differences of adjacent R-R intervals; SDNN, standard deviation of all R-R intervals. Data are presented as median of each subject's overall nights' median*.

### HRV segments during sleep

For the 45 nights included in the analysis, a total of 1792 5-min segments were analyzed (14 in sleep stage 1, 730 in sleep stage 2, 339 SWS and 709 in REM sleep). The low abundance of sleep stage 1 and the generally short duration of this stage resulted in this low number of segments, therefore, only data from sleep stage 2, REM sleep, and SWS were included for further analyses. Mean numbers of analyzed segments per night was 16 for stage 2 (range 3–40), 8 for SWS (range 3–16), and 16 for REM sleep (range 3–24). Of the segments included in the variance analysis, stage 2 segments occurred 265 [IQR 189, 350] min after sleep onset for stage 2, SWS segments 101 [33, 140] min after sleep onset and REM sleep segments after 325 [222, 408] min. Raw data of HF power and LF power over the course of the night in the different sleep stages are presented in the supplementary material (Figure S1).

### Effect of order of the night

In our sample, there was no evidence of an effect of the order of the night on any of the analyzed parameters (all *p* > 0.15).

### Variance partitioning of HRV parameters in the different sleep stages

The between subjects, between nights (within each subject) and between segments (within each night) *SD*s for each individual sleep stage are reported in Table 2. For the calculated variances, the proportion of the total variance as well as of the within-subject variance are reported in Table 3.

**Table 2:** Standard deviations of HRV parameters of the different sleep stages.

	**SWS** ***SD***	**Stage 2** ***SD***	**REM sleep** ***SD***
**HEART RATE**			
Between Subj	5.63	4.85	5.90
Between Nights	2.66	1.91	2.39
Between Segments	1.87	2.05	2.80
**ln(HF POWER)**			
Between Subj	0.88	0.63	0.69
Between Nights	0.20	0.39	0.26
Between Segments	0.33	0.50	0.44
**ln(LF POWER)**			
Between Subj	0.61	0.78	0.56
Between Nights	0.24	0.20	0.14
Between Segments	0.52	0.71	0.49
**ln(LF/HF)**			
Between Subj	0.58	0.62	0.46
Between Nights	0.28	0.37	0.22
Between Segments	0.49	0.63	0.45
**ln(RMSSD)**			
Between Subj	0.44	0.38	0.38
Between Nights	0.10	0.12	0.12
Between Segments	0.17	0.23	0.21
**ln(SDNN)**			
Between Subj	0.36	0.32	0.20
Between Nights	0.08	0.10	0.06
Between Segments	0.20	0.38	0.28

*SD, standard deviation; HF, high frequency; LF, low frequency; ln, natural logarithm; SWS, slow-wave sleep; REM, rapid eye movement*.

**Table 3:** Variance decomposition of total variance and within subject variance, as well as intra-class correlation coefficients (indicated in brackets) of the different sleep stages.

	**SWS**		**Stage 2**		**REM sleep**	
	**Total variance**	**Variance_Within Subjects_**	**Total variance**	**Variance_Within Subjects_**	**Total variance**	**Variance_Within Subjects_**
**HEART RATE**						
Var_Between Subj_ [%]	74.9 (0.75)	NA	75.0 (0.75)	NA	76.8 (0.77)	NA
Var_Between Nights_ [%]	16.8	66.9 (0.67)	11.6	46.2 (0.46)	11.8	42.1 (0.42)
Var_Between Segments_ [%]	8.3	33.1	13.4	53.8	16.2	57.9
**ln(HF POWER)**						
Var_Between Subj_ [%]	83.7 (0.84)	NA	50.0 (0.50)	NA	64.9 (0.65)	NA
Var_Between Nights_ [%]	4.3	26.6 (0.27)	18.8	37.5 (0.38)	9.5	26.9 (0.27)
Var_Between Segments_ [%]	12.0	73.4	31.2	62.5	25.7	73.1
**ln(LF POWER)**						
Var_Between Subj_ [%]	52.8 (0.53)	NA	52.6 (0.53)	NA	54.4 (0.54)	NA
Var_Between Nights_ [%]	8.6	18.2 (0.18)	3.4	7.3 (0.07)	3.5	7.7 (0.08)
Var_Between Segments_ [%]	38.5	81.8	44.0	92.7	42.1	92.3
**ln(LF/HF)**						
Var_Between Subj_ [%]	51.5 (0.52)	NA	41.9 (0.42)	NA	45.7 (0.46)	NA
Var_Between Nights_ [%]	12.1	25.0 (0.25)	15.0	25.9 (0.26)	10.9	20.0 (0.20)
Var_Between Segments_ [%]	36.4	75.0	43.0	74.1	43.5	80.0
**ln(RMSSD)**						
Var_Between Subj_ [%]	83.8 (0.84)	NA	68.8 (0.69)	NA	70.9 (0.71)	NA
Var_Between Nights_ [%]	4.3	26.3 (0.26)	7.00	22.4 (0.22)	7.5	25.9 (0.26)
Var_Between Segments_ [%]	11.9	73.7	24.2	77.6	21.6	74.1
**ln(SDNN)**						
Var_Between Subj_ [%]	73.4 (0.73)	NA	39.6 (0.40)	NA	33.1 (0.33)	NA
Var_Between Nights_ [%]	3.4	12.8 (0.13)	3.9	6.5 (0.07)	3.3	4.9 (0.05)
Var_Between Segments_ [%]	23.2	87.2	56.5	93.5	63.6	95.1

*Note that the percentage of explained between-subject variance is equivalent to the between-subjects/within-subjects ICC, and the between-night variance in percentage of the within-subject variance is equivalent to the between-nights/within-nights ICC*.

For all HRV parameters in all sleep stages and all HRV parameters, with exception of LF/HF ratio and SDNN in stage 2 and in REM sleep, the largest proportion of total variance was explained by the between-subject variance (84% for HF power and RMSSD in SWS). The between segment variances were small in SWS, in particular for heart rate, HF power, and RMSSD, where the between segment variances were between 8 and 12% of the total variance. On the other hand, the between-segment variance was around 40% of the total variance for LF power and LF/HF ratio in all sleep stages and >56% for SDNN in stage 2 and REM.

### ICC

The between-subjects/within-subjects ICC is equivalent to the percentage of explained between-subject variance (Table 3). The between-nights/within-nights ICC is equivalent to the between-night variance of the within-subject variance (Table 3). In accordance to between subject variances, between-subjects/within-subjects ICCs were good for heart rate, HF power, and RMSSD in SWS. Good to excellent between-subjects/within-subjects ICCs were observed in all sleep stages for heart rate and RMSSD with the highest values in SWS. For LF power and LF/HF ratio, fair ICCs were observed with comparable values in all analyzed sleep stages. Between-nights/within-nights ICCs were generally poor, except for heart rate, where it was good for SWS and fair for stage 2 and REM sleep.

### Verification of the identified SWS segment (segment_Low rRR_)

Of all analyzed nights, 87% (39 out of 45) of the detected segments were fully located within SWS and where thus correctly identified. Of the remaining 6 segments, 2 were partially located during SWS (>50%), 2 segments were located completely in stage 2 and 2 segments were located across stage 1 and stage 2. An example of the sleep staging of a typical night and the rRR, used to identify the Segment_low rRR_, of the same night are shown in Figure 1.

## Discussion

In the present study aiming at characterizing the reproducibility of HRV variables in the different sleep stages, we found better reproducibility (between-subject/within subject ICC and SDs) for HF power, RMSSD, and heart rate during SWS compared to the other sleep stages. In all sleep stages, variances between subjects generally accounted for the highest percentage of total variance, followed by between-segment variance (within same nights) and between nights within each subject. Between-subject/within subject ICCs were excellent for heart rate in all sleep stages. They were also excellent for HF power and RMSSD and good for SDNN in SWS. Fair ICCs were found for LF and LF/HF ratio in all sleep stages. When a single 5-min segment was located within SWS identified by an algorithm based on HRV data only, 87% were correctly placed within SWS according to PSG.

The variances observed during SWS were comparable to the variances reported by Schroeder et al. (2004) for HRV measurements in wake supine position, using a repeated measure study design similar to ours. The lowest residual variance (within-night variance), reflecting the best reproducibility, was found for heart rate and HF power in SWS, followed by stage 2, and highest residual variance and poorest reproducibility in REM sleep. These results are in accordance with the low cardiovascular variability reported during SWS (Franzini, 2000). Thus, while heart rate, HF power and RMSSD in SWS are highly reproducible under comparable conditions, it remains open whether it is also a stage suited to detect acute or chronic stress. Acute psychological stress was found to be reflected by lower HF power in stress situations both during NREM and REM sleep, and of note, with the difference increasing over the course of the night (Hall et al., 2004). Further research is required to assess how well HRV parameters measured in the different sleep stages may reflect the effects of different stressors.

This is, to the best of our knowledge, the first study to quantify variance components of HRV parameters as well as reproducibility in different sleep stages. Reproducibility of HRV measurements in an awake state between different days has been extensively studied (Schroeder et al., 2004; Pinna et al., 2007; Sacre et al., 2012; Schafer et al., 2015; Silva et al., 2017). During sleep, reproducibility of mean heart rate of a whole night's sleep has been investigated (Waldeck and Lambert, 2003). In these studies, ICC, a measure for relative reproducibility, for HRV measurements between different days ranged from 0.64 to 0.91 when measured in a wake state in supine position. Similar values of reproducibility were found for different HRV parameters in the studies cited above. In our study, ICCs were best for HF power (0.84), RMSSD (0.84), and SDNN (0.73) in SWS, and poorer for the two other sleep stages. For heart rate ICCs were between 0.75 and 0.77 in all sleep stages. For LF power and LF/HF power ratio ICCs were below 0.55 in all sleep stages. The low ICC for LF power and LF/HF ratio during SWS may have been a consequence of the generally very low values of LF power in all subjects, reflecting low sympathetic activity (Somers et al., 1993; Silvani and Dampney, 2013) during deep sleep. Greater heterogeneity of the population regarding the parameter of interest automatically leads to greater ICCs compared to more homogeneous populations. The generally poorer ICCs in stage 2 and REM sleep for all analyzed HRV parameters may be explained by the higher frequency and higher amplitude of sympathetic surges and therefore the lower stationarity of HRV parameters in these two stages.

Based on the results of our study, it seems advisable to assess HF power and/or RMSSD during SWS rather than another sleep stage due to a smaller between segment variance. Given that the variance was greater between segments than between nights (and this was not due to a trend over the course of the night), it seems important to average over several segments within one night. Our findings of a low between-subject/within-subject ICC for LF power and LF/HF ratio in all sleep stages questions the suitability of sleep in general for the assessment of these parameters, which are often used as marker of sympathetic activity.

In previous studies assessing the effects of sleep stages on heart rate and HRV, the highest values of heart rate were observed during REM sleep and lower heart rates during stage 2 and SWS with no significant difference between the latter two stages (Trinder et al., 2001; Boudreau et al., 2013). Similar effects of sleep stages on heart rate were observed in our study. HF power was comparable in SWS, stage 2 and REM sleep which was also the case in previous studies (Busek et al., 2005; Boudreau et al., 2013) while others have observed a reduction in HF power in REM sleep compared to the other sleep stages (Trinder et al., 2001). In our study, LF power was lowest during SWS and highest during REM sleep. This is in accordance with results from other studies and indicates a clear sleep stage dependence of LF power (Trinder et al., 2001; Busek et al., 2005; Cabiddu et al., 2012; Boudreau et al., 2013). Reported differences between sleep stages with regard to absolute LF power (up to 140%) in our and previous studies were much bigger than with regard to absolute HF power (<25%). The large differences in LF power between different sleep stages resulted in a concomitantly large difference in LF/HF ratio, being lowest in SWS, followed by stage 2 and being highest during REM sleep. These findings are also in accordance with previous studies (Scholz et al., 1997; Elsenbruch et al., 1999; Ferri et al., 2000).

Many studies reported a rapid decrease in heart rate concomitant with the wake-sleep transition (Shinar et al., 2006) and a more gradual decrease thereafter throughout the night (Burgess et al., 1999). This has been clearly shown when the different sleep stages were analyzed separately (Cajochen et al., 1994; Trinder et al., 2001). When heart rate is analyzed irrespective of the sleep stages, then often an increase in heart rate toward the morning hours was observed. Reasons for this may be the higher abundance of REM sleep in the later part of sleep, the circadian increase in cortisol concentration in the blood and/or the increase in body temperature toward the morning (Burgess et al., 1997). Further, heart rate has been found to steeply increase at the end of each SWS phase, when an arousal and the concomitant blood pressure surge initiates a transition into REM sleep (Bonnet and Arand, 1997). These autonomic processes precede the central activities according to which the sleep stages are classified. Unexpectedly, in our study, a mean increase in heart rate of 3.2 ± 0.4 (*SD*) bpm was observed in the first SWS phase. This was followed by a mean decrease in heart rate of (2.9 ± 0.5 bpm) over the rest of the night. The initial increase in heart rate in the first SWS phase has not been reported previously, however, some data seems to be in agreement with our finding (Cajochen et al., 1994; Burgess et al., 1999; Brandenberger et al., 2005). This acceleration of heart rate in the first SWS phase was not accompanied by a concomitant decrease in HF power as would be expected when caused by a decreased vagal activity or by an increased sympathetic activity. We suspect factors other than the CANA to be responsible for this discrepancy.

The accuracy of our algorithm for the detection of a segment in SWS by relying solely on HRV data was satisfactory with most of the segments (87%) being accurately placed within a phase of SWS (as verified by PSG). Different HRV based algorithms to perform sleep staging have been proposed and compared to sleep staging by PSG. Most algorithms have used HRV frequency domain parameters (Shinar et al., 2001; Mendez et al., 2009). Other studies have used a combination of different HRV parameters (time domain, frequency domain, non-linear parameters) with the aim to improve the precision of their classification algorithm (Xiao et al., 2013; Fonseca et al., 2015; Yoon H. et al., 2017; Yoon H. N. et al., 2017). Compared to the traditional sleep staging by PSG, an accuracy of 69% in the classification of sleep stages was achieved in the study by Fonseca et al. (2015). In an attempt to classify sleep into wake-REM-NREM sleep, an accuracy of 79% has been reported using a combination of frequency domain parameters (Mendez et al., 2009). In one early study (Shinar et al., 2001), the use of HRV (LF/HF ratio) to identify SWS resulted in 80% correct identification of SWS. Recently, using HRV parameters besides respiratory signals measured by respiratory inductance plethysmography, an accuracy of 89% was reported for the detection of slow wave sleep (Long et al., 2017). Similarly, using a combination of time domain, frequency domain and non-linear parameters resulted in an Cohens kappa of 0.56 for the detection of SWS (Yoon H. N. et al., 2017). Combining HRV frequency domain parameters with actigraphy resulted in an accuracy of 74% (Muzet et al., 2016), comparable to the methods based on HRV only. In contrast to these studies, our aim was not to perform an actual sleep staging but to verify that a segment identified by HRV analysis was located within SWS This explains the rather high accuracy of our algorithm, based on rRR values, a simple HRV measure. In one of our previous studies in elite athletes (Herzig et al., 2016), the same algorithm resulted in a yield of 75% of segments placed in SWS according to non-conventional PSG sleep staging with a mobile 3-lead EEG system.

A limitation of the present study was the absence of a direct measurement of the CANA. However, the aim of this study was to quantify the reproducibility of HRV parameters without making direct inferences on the CANA. Further, we have only quantified reproducibility under standardized conditions, it will be the task of future studies to assess the sensitivity of HRV parameters in different sleep stages to detect disturbances, such as psychological or physical acute or chronic stress. While cortical activity largely follows autonomic activity (Jurysta et al., 2003) some dissociation may occur. Further, data of the present study was collected for a different study assessing a treatment effect. However, the small between nights variance support the absence of a treatment or night order effect. Last but not least, the present study was conducted in healthy subjects, and findings cannot be extrapolated to diseased populations. Strengths of the present study are the statistical analysis of the nested design separating variances due to different factors. This is the first study to assess the reproducibility of HRV parameters during sleep and to compare reproducibility of these parameters between the different sleep stages. The simple algorithm to identify SWS used in the present study will allow wide usage of this efficient method to measure HRV parameters in SWS.

## Conclusion

In conclusion, SWS offers a stationary and reproducible phase for determination of heart rate, HF power and RMSSD, and can satisfactorily be detected based on R-R intervals derived from ECG recordings, without the need of full PSG. LF power and LF/HF ratio had fair to poor reproducibility in all sleep stages. Between segment variance was high compared to between night variance for all HRV parameters except heart rate, indicating that averaging over segments within each night is advisable.

## Author contributions

XO, RR, and PA designed the experiment and conducted the data collection. DH, PE, MW, XO, and PA were involved in data analysis. DH and PE composed the manuscript. All authors reviewed and approved the manuscript.

### Conflict of interest statement

The authors declare that the research was conducted in the absence of any commercial or financial relationships that could be construed as a potential conflict of interest. The reviewer MWR and handling Editor declared their shared affiliation.

## Supplementary material

Figure S1 HF power **(Left)** and LF power **(Right)** of 5-min segments over the course of the night of all subjects and all nights. The different colors represent data of different subjects and segments corresponding to the same night are connected by a solid line. The segments of the pooled sleep stages are shown in the top panel, time course of the segments of the different sleep stages are shown in the lower panels. Time 0 corresponds to sleep onset.
