# Supplementary figures and images for: Reproducibility of Heart Rate Variability Is Parameter and Sleep Stage Dependent
Source: Front Physiol. 2018 Jan 10;8:1100. doi: 10.3389/fphys.2017.01100 (PMC5767731; doi:10.3389/fphys.2017.01100)

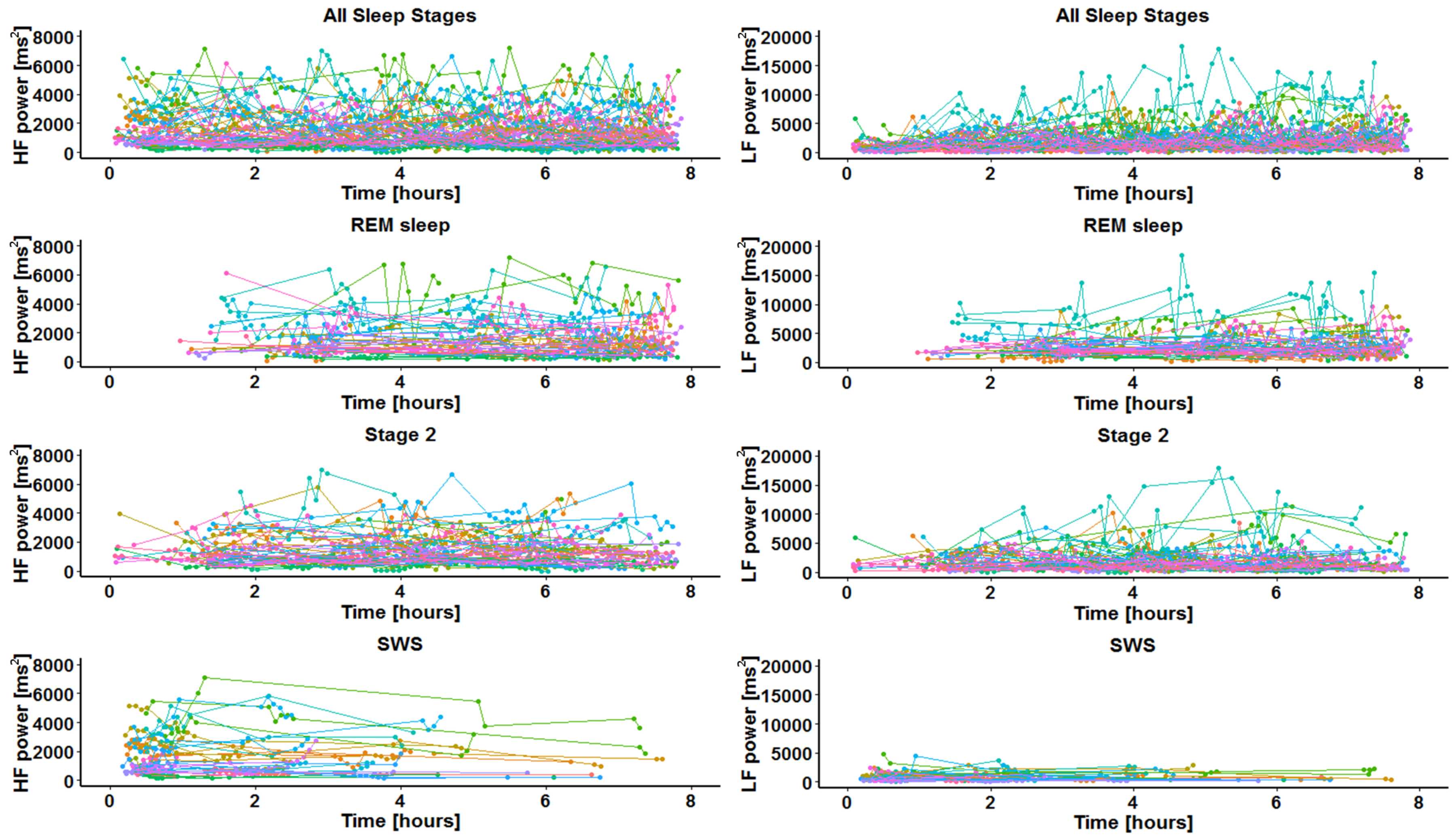

Supplement: Figure S1 — HF power (Left) and LF power (Right) of 5-min segments over the course of the night of all subjects and all nights. The different colors represent data of different subjects and segments corresponding to the same night are connected by a solid line. The segments of the pooled sleep stages are shown in the top panel, time course of the segments of the different sleep stages are shown in the lower panels. Time 0 corresponds to sleep onset. [file Image1.TIFF]
